# Metagenomic evidence for antibiotic-associated actinomycetes in the Karamay Gobi region

**DOI:** 10.3389/fmicb.2024.1330880

**Published:** 2024-03-05

**Authors:** Shuai Yang, Wei Zhang, Bo Yang, Xin Feng, Yiyang Li, Xiaolin Li, Qin Liu

**Affiliations:** ^1^Xinjiang Second Medical College, Xinjiang, China; ^2^Key Laboratory of Protection and Utilization of Biological Resources in Tarim Basin Co-funded by Xinjiang Production & Construction Corps and The Ministry of Science & Technology, Tarim University, Alar, China

**Keywords:** desert soil, metagenomic sequencing, bioinformatics, actinomycetes, antibiotic

## Abstract

Due to the misuse of antibiotics, there is an increasing emergence and spread of multidrug-resistant (MDR) bacteria, leading to a human health crisis. To address clinical antibiotic resistance and prevent/control pathogenic microorganisms, the development of novel antibiotics is essential. This also offers a new approach to discovering valuable actinobacterial flora capable of producing natural bioactive products. In this study, we employed bioinformatics and macro-genome sequencing to collect 15 soil samples from three different locations in the Karamay Gobi region. First, we assessed the diversity of microorganisms in soil samples from different locations, analyzing the content of bacteria, archaea, actinomycetes, and fungi. The biodiversity of soil samples from outside the Gobi was found to be higher than that of soil samples from within and in the center of the Gobi. Second, through microbial interaction network analysis, we identified actinomycetes as the dominant group in the system. We have identified the top four antibiotic genes, such as *Ecol_fabG_TRC*, *Efac_liaR_DAP*, *tet*A (58), and *mac*B, by CARD. These genes are associated with peptide antibiotics, disinfecting agents and antiseptics, tetracycline antibiotics, and macrolide antibiotics. In addition, we also obtained 40 other antibiotic-related genes through CARD alignment. Through in-depth analysis of desert soil samples, we identified several unstudied microbial species belonging to different families, including *Erythrobacteriaceae*, *Solirubrobacterales*, *Thermoleophilaceae*, *Gaiellaceae*, *Nocardioidaceae*, *Actinomycetia*, *Egibacteraceae*, and *Acidimicrobiales*. These species have the capability to produce peptide antibiotics, macrolide antibiotics, and tetracycline antibiotics, as well as disinfectants and preservatives. This study provides valuable theoretical support for future in-depth research.

## 1 Introduction

The increase in antibiotic resistance in pathogenic bacteria poses a significant and global threat to public health. Reliable reports have shown that antimicrobial resistance (AMR) is associated with 700,000 deaths per year worldwide (Fisher et al., 2022). Antibiotics are widely used in human healthcare, veterinary medicine, and agriculture, leading to their continuous release into the environment (Su and Wen, 2022). As a result, antibiotic resistance genes (ARGs) have been introduced into various settings, including clinical facilities (hospitals and clinics), veterinary facilities, the human gastrointestinal microbiome, wastewater treatment plants, and the natural environment (soil, water, and air) (Hart et al., 2023). ARGs caused by antibiotic resistance are considered a major challenge in modern medicine, making the effective clinical treatment of bacterial infections difficult. Ongoing research by scientists aims to address this issue. In Dr. Su’s research, 36 high-risk ARGs that may threaten public health were identified, along with priority carriers of ARGs such as *Escherichia coli*, *Enterococcus faecalis*, and *Pseudomonas influenzae* type A. Metagenomics and bioinformatics analyses revealed 15 types of ARGs with high abundance, including 381 ARG subtypes, in a drinking water treatment plants (DWTPs) (Gu et al., 2023). Although urban sewage is effectively treated in WWTPs before discharge, combined sewer overflows (CSOs) caused by storms can directly release significant amounts of ARGs into the environment (Jang et al., 2021). The research conducted on the antibiotic resistome in the CSOs has revealed that the flow of antibiotics and host range take place at the allele level, indicating that the study of mobile genetic elements (MGEs) warrants greater attention from scientific researchers (Zhang et al., 2022).

Antibiotics are a class of chemicals that kill or inhibit bacteria. They achieve bactericidal or bacteriostatic effects by interfering with the synthesis of bacterial cell walls, changing the internal metabolism of bacteria, and interfering with the synthesis of bacterial nucleic acids, thus treating various bacterial infections (Corno et al., 2014). However, despite being one of the greatest discoveries of our time, the use of antibiotics to treat bacterial infections also presents some challenges. First, bacterial resistance is gradually increasing, rendering some antibiotics less effective or even ineffective. One possible explanation for this is that sometimes a single resistance allele mechanism can confer resistance to several different antibiotics, including antibiotics of different classes (Allen et al., 2019). Second, non-pathogenic bacteria can transform into drug-resistant strains by acquiring resistance genes. Since the introduction of penicillin antibiotics in the 1940s, antibiotic research has focused on discovering and modifying antibiotic structures, as well as utilizing enhancers and accelerators to aid in fighting bacterial infections. Bacteria produce antimicrobial substances that kill or inhibit competitors accordingly. Previous observations have indicated that natural products (NPs) serve as a significant reservoir of novel antibiotics. In a study conducted by Mr. Wang, it was demonstrated that ρ-aminobenzoic acid encoding BGCs, cloned from soil metagenomes, have played a crucial role in guiding the synthesis of albicidin and cystobactamid analogs (Wang et al., 2021). Another investigation explored the ocean as a potential source for bacterial isolation, aiming to discover new secondary metabolites (Purves et al., 2016). Metagenomics analysis of seawater samples collected from various depths in Antarctica and the Arctic reveals the presence of microorganisms capable of producing primary metabolites. These microorganisms primarily belong to *Actinobacteria*, *Chloroflexi*, *Proteobacteria*, *Acidobacteria*, and two *Candidate phyla*, AD3 and WPS-2 (Cao et al., 2020). The microbial bioactive metabolites produced by Actinomycetales account for 45% of the total identified compounds. Among these, the genus streptomyces alone is responsible for 75% of these metabolites (Bérdy, 2005). Consequently, numerous researchers have focused on isolating, identifying, and preserving Actinomyces strains. In our research group, we utilized Gao’s medium to isolate and identify Actinomyces, resulting in the acquisition of dozens of *Streptomyces* strains. However, one limitation of this method is the high proportion of strains obtained through repeated isolation. Previous studies have demonstrated that metagenomes have emerged as the most effective approach for investigating bacterial communities and their microbial bioactive metabolites. Existing research suggests that the metagenome assembly of DWSS samples can provide insights into the microbial community, antibiotic resistome, and MGEs co-existing with ARGs and ARG hosts (Ke, et al., 2023).

The Karamay Gobi region is a typical extreme environment characterized by a large temperature difference between day and night, high radiation, and low precipitation. Actinomycetes found in this environment exhibit unique characteristics compared to those found in ordinary environments. These distinct actinomycete resources have the potential to be a valuable source for the discovery of new structural antibiotics. The objective of this project is to explore the characteristics of actinomycetes in extreme environments, analyze the distribution of antibiotic genes, and provide theoretical guidance for the future isolation of actinomycetes. First, the dominant flora in the soil was analyzed using metagenome sequencing to identify the prevalent flora in this environment. Then, functional genes and species related to antibiotics were analyzed at the functional level to speculate on the potential presence of secondary actinomycetes. These metabolites serve as a foundation for designing specialized media and targeting the isolation of active substances in the future.

## 2 Materials and methods

### 2.1 Soil sample collection

**Figure 1:**
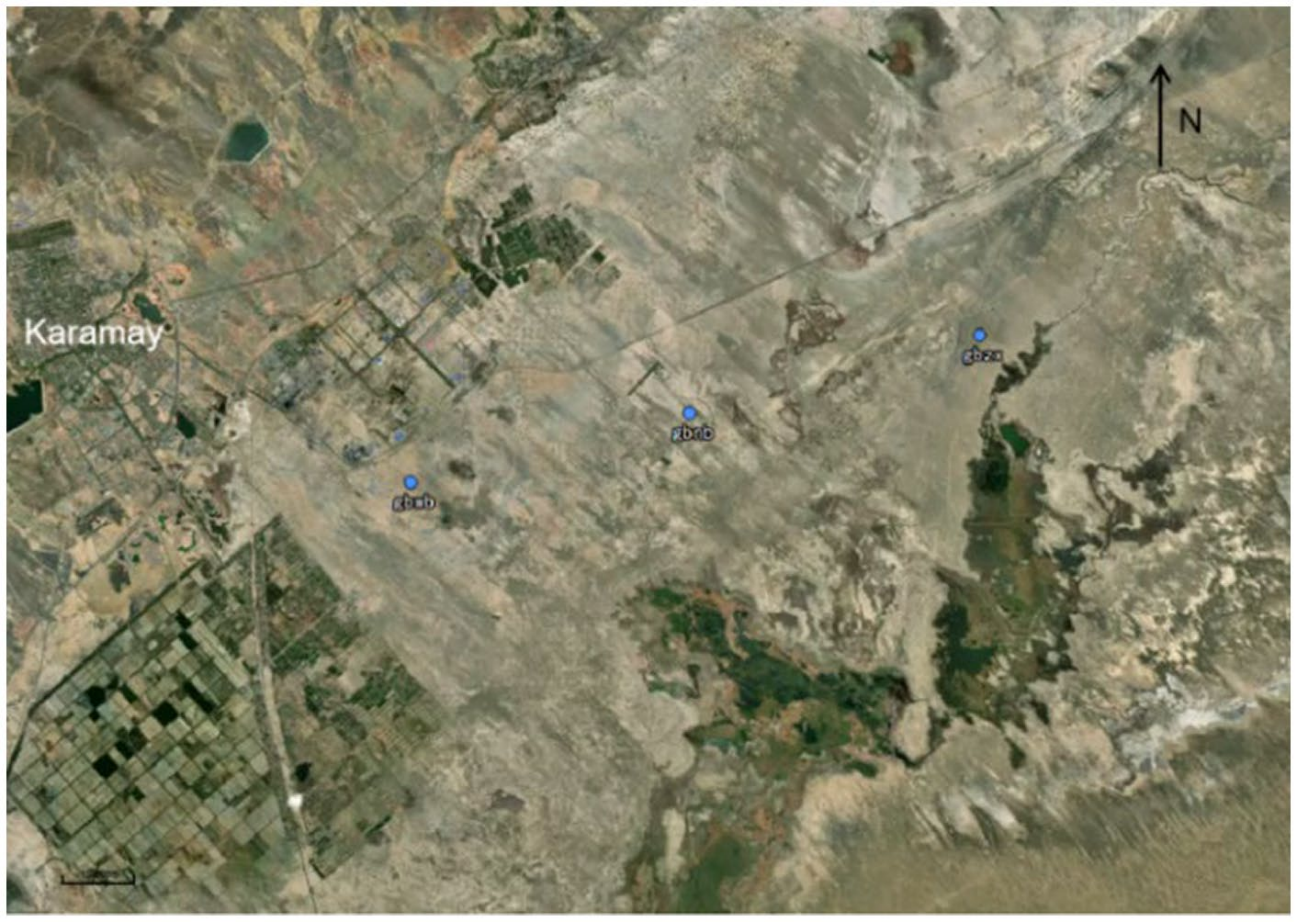
Sampling points were collected in the Karamay Gobi region. The locations of the samples are represented by blue dots and labels. The sampling method used was the ‘five-point sampling method’ (Wang et al., 2020). This method involved determining the central sampling point within a 1-kilometer radius as the midpoint of the diagonal line. Then, four points equidistant from the central sampling point were selected on the diagonal line as additional sampling points. A total of five samples were collected from each location, resulting in a total of 15 sample points (Xiao et al., 2022).

The sampling sites were distributed in the Karamay Gobi region between 84°44′ and 86°1′ E longitude and 44°7′ and 46°8’ N latitude (Figure 1). The impact of the unique desert ecosystem on climate change and biodiversity in this region has always been a focal point for researchers. For sampling, we selected the outer edge, inner edge, and central part of the Karamay Gobi Region, with each sampling site separated by approximately 5 km. The labeling scheme used is as follows: the outer samples (W1–W5) correspond to the gbwb group, the inner samples (N1–N5) correspond to the gbnb group, and the central samples (Z1–Z5) correspond to the gbzx group.

### 2.2 DNA extraction and Illumina Nova_Seq sequencing

The OMEGA Mag-Bind Soil DNA Kit (M5635-02) (Omega Bio-Tek, Norcross, GA, United States) was used to extract the total microbial genomic DNA from soil samples. A Qubit™ 4 Fluorometer (Qubit™ Assay Tubes: Q32856; Qubit™ 1X dsDNA HS Assay Kit: Q33231) (Invitrogen, United States) and agarose gel electrophoresis were used to measure the quantity and quality of extracted DNA, respectively. Then, the qualified microbial DNA was processed to construct metagenome shotgun sequencing libraries with the Illumina TruSeq Nano DNA LT Library Preparation Kit. Each eligible PE150 library was sequenced with the Illumina NovaSeq platform (Illumina, USA) at Personal Biotechnology Co., Ltd. (Shanghai, China).

### 2.3 Metagenome analysis

High-quality reads (clean reads) were obtained by removing sequencing adapters from sequencing reads with Cutadapt (v1.2.1) (Martin, 2011) and trimming low-quality reads with fastp (Chen et al., 2018). Then, taxonomical classifications of metagenomics sequencing reads from each sample were performed using Kaiju (1.9.0)^1^ (Menzel et al., 2016) against a RefSeq-derived database, which included genomes from archaea, bacteria, viruses, fungi, protozoans, metazoans, and viridiplantae. To assemble these high-quality reads into contigs, MEGAHIT (v1.1.2) (Li et al., 2015) was used with the meta-large presetted parameters. The generated contigs (longer than 300 bp) were pooled together and clustered using mmseqs2 (Steinegger and SöDing.,2017) with “easy-linclust” mode, setting the sequence identity threshold to 0.95 and covering residues of the shorter contig to 90%. Compared to diamond, mmseqs2 offers the advantage of a lower error rate (Hernández-Salmerón and Moreno-Hagelsieb, 2020). The non-redundant contigs were obtained by aligning them against the NCBI-nt database by mmseqs2 (Steinegger and SöDing, 2017) in “taxonomy” mode, and contigs assigned to Viridiplantae or metazoa were dropped in the following analysis. The prediction of the genes in the contigs was performed with MetaGeneMark (Zhu et al., 2010). The functionality of the non-redundant genes was annotated by adopting mmseqs2 (Steinegger and SöDing, 2017) with the “search” mode against the CARD (v3.2.4)^2^ protein databases. To assess the abundances of these genes, the high-quality reads from each sample were mapped onto the predicted gene sequences using salmon (Patro, 2015) in the quasi-mapping-based mode with “--meta --minScoreFraction = 0.55,” and the TPM (Transcripts Per Kilobase per Million mapped reads) was used to normalize abundance values in metagenomes.

## 3 Results

### 3.1 Soil characteristics analysis

The results of soil physicochemical property measurements demonstrated notable variations among sampling sites in the Gobi region for all soil physicochemical properties, except for pH. It should be noted that the variability of pH was comparatively lower, as shown in Table 1. In terms of the experimental results, the levels of organic matter, soluble salts, fast-acting potassium, and phosphate were higher in the Gobi region compared to the other two regions. We hypothesized that the soluble salts may contain growth factors that promote the growth of actinomycetes. Therefore, if we want to isolate actinomycetes in this region, it is necessary to add additional soluble salts to the basal culture medium. On the other hand, the total nitrogen and organic carbon species were more abundant in the interior of the Gobi, indicating that the soil in the central part of the Gobi is nutrient-poor.

**Table 1:** Soil physical and chemical properties of the Karamay Gobi Desert.

Soil physical and chemical properties of the Karamay Gobi Desert							
No.	Name	pH	organic carbon (g/kg)	organic matter(OM)(g/kg)	total nitrogen(TN) (g/kg)	soluble salts(g/kg)	fast-acting potassium (mg/kg)
1	GBZX	7.16	5.45	9.39	0.45	13.00	158.32
2	GBWB	7.49	7.73	13.34	0.65	23.88	175.68
3	GBNB	7.29	8.97	15.47	0.80	15.76	146.99

### 3.2 Microbial diversity analysis

**Figure 2:**
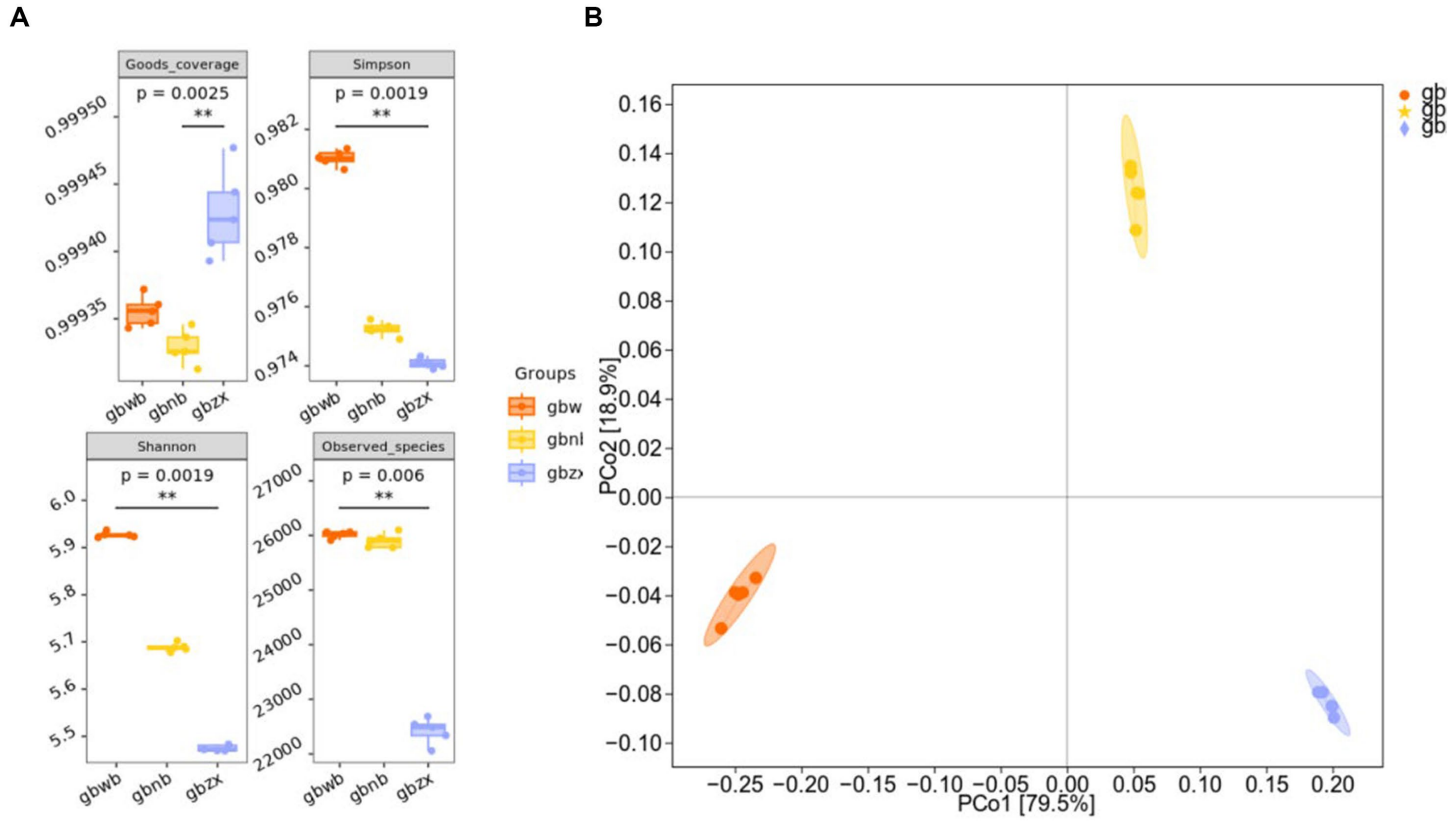
Microbial diversity analysis. **(A)** Analysis of alpha diversity box plot (Kruskal–Wallis rank-sum test and Dunn’s test were used as post-tests) among groups. **(B)** Analysis of beta diversity (principal coordinates analysis using the Bray–Curtis dissimilarity) among groups.

Microbial alpha diversity indices, including Chao (1984) and Shannon (1948a,b), and ACE, were used to measure species diversity complexity, richness, and sample coverage (Chao and Lee, 1992). As depicted in Figure 2A, significant differences were observed in the ACE and Chao1 indices between the gbnb group and the gbwb and gbzx groups, while a significant difference in the Shannon index was found between the gbwb and gbzx groups. Notably, the Chao1 and ACE indices of the gbzx group were the lowest, indicating a relatively lower actual species number and species richness compared to the other two groups. The gbwb group exhibited the highest Shannon value, while the gbzx group had the lowest, suggesting that the community diversity of the three groups followed the order of gbwb group, gbnb1, and gbzx from high to low. The species distribution in the gbwb group was more uniform (Supplementary Table S1). Principal coordinate analysis using the Bray–Curtis dissimilarity (Bray and Curtis, 1957) also demonstrated significant differences in the microbiota composition among the three groups (analysis of similarities; ADONIS test; R2-statistic = 0.9826, *p* = 0.001) (Figure 2B).

### 3.3 Microbial composition analysis

**Figure 3:**
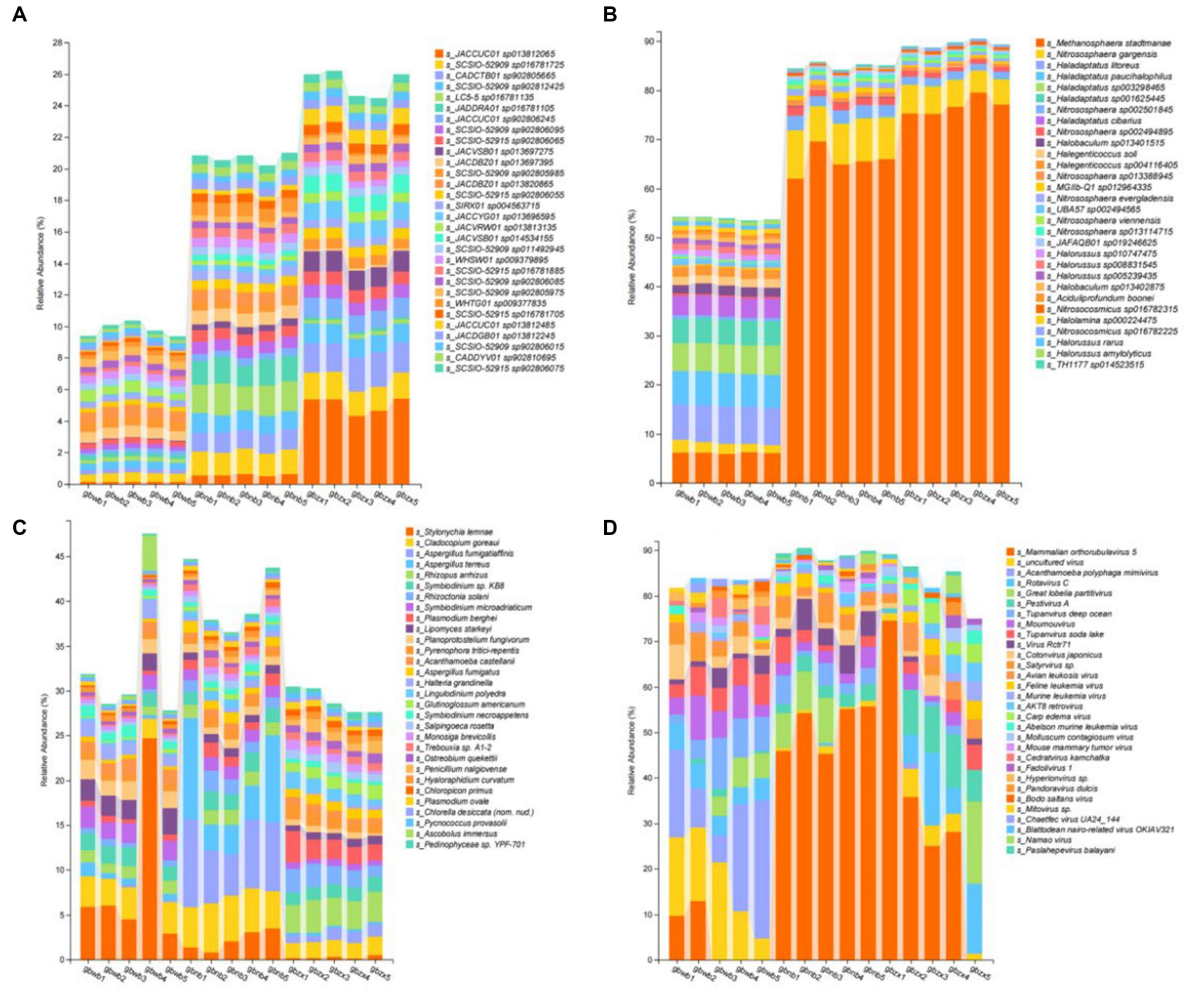
Bar chart reporting the composition of dominant species. **(A)** The top 30 species in relative abundance at the species level in bacterial communities. **(B)**The top 30 species in Relative abundance at the species level in archaea communities. **(C)** The top 30 species in relative abundance at the species level in Eukaryon communities. **(D)** The top 30 species in relative abundance at the species level in virus communities.

The dominant bacterial species in all soil samples were *SCSIO-52915* sp902806075, *CADDYV01* sp902810695, *SCSIO-52909* sp902806015, *JACDGB01* sp013812245, *JACCUC01* sp013812485, *SCSIO-52915* sp016781705, *WHTG01* sp009377835, *SCSIO-52909* sp902805975, *SCSIO-52909* sp902806085, *SCSIO-52915* sp016781885, *WHSW01* sp009379895, *SCSIO-52909* sp011492945, *JACVSB01* sp014534155, *JACVRW01* sp013813135, *JACCYG01* sp013696595, *SIRX01* sp004563715, *SCSIO-52915* sp902806055, *JACDBZ01* sp013820865, *SCSIO-52909* sp902805985, *JACDBZ01* sp013697395, *JACVSB01* sp013697275, *SCSIO-52915* sp902806065, *SCSIO-52909* sp902806095, *JACCUC01* sp902806245, *JADDRA01* sp016781105, *LC5-5* sp016781135, *SCSIO-52909* sp902812425, *CADCTB01* sp902805665, *SCSIO-52909* sp016781725, and *JACCUC01* sp013812065 (Figure 3A);

The dominant archaea species in all soil samples were *TH1177* sp014523515, *Halorussus amylolyticus*, *Halorussus rarus*, *Nitrosocosmicus* sp016782225, *Halolamina* sp000224475, *Nitrosocosmicus* sp016782315, *Aciduliprofundum boonei*, *Halobaculum* sp013402875, *Halorussus* sp005239435, *Halorussus* sp008831545, *Halorussus* sp010747475, *JAFAQB01* sp019246625, *Nitrososphaera* sp013114715, *Nitrososphaera viennensis*, *UBA57* sp002494565, *Nitrososphaera evergladensis*, *MGIIb-Q1* sp012964335, *Nitrososphaera* sp013388945, *Halegenticoccus* sp004116405, *Halegenticoccus soli*, *Halobaculum* sp013401515, *Nitrososphaera* sp002494895, *Haladaptatus cibarius*, *Nitrososphaera* sp002501845, *Haladaptatus* sp001625445, *Haladaptatus* sp003298465, *Haladaptatus paucihalophilus*, *Haladaptatus litoreus*, *Nitrososphaera gargensis*, and *Methanosphaera stadtmanae* (Figure 3B).

The dominant Eukaryon species in all soil samples were *Pedinophyceae* sp. YPF-701, *Ascobolus immersus*, *Pycnococcus provasolii*, *Chlorella desiccata* (nom. Nud.), *Plasmodium ovale*, *Chloropicon primus*, *Hyaloraphidium curvatum*, *Penicillium nalgiovense*, *Ostreobium quekettii*, *Trebouxia* sp. A1-2, *Monosiga brevicollis*, *Salpingoeca rosetta*, *Symbiodinium necroappetens*, *Glutinoglossum americanum*, *Lingulodinium polyedra*, *Halteria grandinella*, *Aspergillus fumigatus*, *Acanthamoeba castellanii*, *Pyrenophora tritici-repentis*, *Planoprotostelium fungivorum*, *Lipomyces starkeyi*, *Plasmodium berghei*, *Symbiodinium microadriaticum*, *Rhizoctonia solani*, *Symbiodinium* sp. KB8, *Rhizopus arrhizus*, *Aspergillus terreus*, *Aspergillus fumigatiaffinis*, *Cladocopium goreaui*, and *Stylonychia lemnae* (Figure 3C).

The dominant virus species in all soil samples were *Paslahepevirus balayani*, *Namao virus*, Blattodean nairo-related virus OKIAV321, Chaetfec virus UA24_144, *Mitovirus* sp., *Bodo saltans virus*, *Pandoravirus dulcis*, *Hyperionvirus* sp., Fadolivirus 1, *Cedratvirus kamchatka*, *Mouse mammary tumor virus*, *Molluscum contagiosum virus*, *Abelson murine leukemia virus*, *Carp edema virus*, AKT8 retrovirus, *Murine leukemia virus*, *Feline leukemia virus*, *Avian leukosis virus*, *Satyrvirus* sp., *Cotonvirus japonicus*, Virus Rctr71, *Tupanvirus soda lake*, *Moumouvirus*, Tupanvirus deep ocean, Pestivirus A, Great lobelia partitivirus, Rotavirus C, *Acanthamoeba polyphaga* mimivirus, uncultured virus, and Mammalian orthorubulavirus 5 (Figure 3D).

We analyzed the data down to the species level. In the bacterial part, it was observed that *JACCUC01* sp013812065 showed significant variation in the samples. The analysis revealed that this bacterium had a high percentage in gbzx, but very little in gbwb and gbnb. However, *JADDRA01* sp016781105 and *LC5-5* sp016781135 exhibited a high percentage in gbnb, but very little in gbwb and gbzx. The distribution of *JACCUC01* sp902806245, *JACCUC01* sp902806245, *JACVSB01* sp013697275, and *JACCYG01* sp013696595 is characterized by gbwb being smaller than gbnb, which in turn is smaller than gbzx. In the archaeal fraction, *Methanosphaera stadtmanae* is the most prevalent species in the Gobi ecosystem. We observed its presence in all three samples we collected, with a particularly high representation in gbzx. On the other hand, *Haladaptatus litoreus*, *Haladaptatus paucihalophilus*, *Haladaptatus* sp003298465, and *Haladaptatus* sp001625445, which are archaea that are abundant in gbwb, show a significant decrease in abundance in both gbnb and gbzx samples. In the eukaryotic section, Stylonychia lemna is found in all parts of the Gobi region. The occurrence of gbwb and gbnb is higher than gbzx. However, it is important to note that the data from gbwb-4 samples are abnormal and should not be considered when comparing the characteristics of this species. *Additionally*, *Aspergillus fumigatiaffinis* and *Aspergillus terreus* are two eukaryotes that are primarily found in gbnb and occur less frequently in gbwb and gbzx. During the analysis of this module, we observed significant fluctuations in the virus section. This variability is likely attributed to the fact that viral reproduction relies on parasitizing living cells and exhibits strong host specificity. To conduct a more comprehensive study on viral diversity in the Gobi region, it might be necessary to collect additional replicates of the samples. From the data, we identified several common virus species in each region, including *Mammalian orthorubulavirus* 5, *Pestivirus* A, *Tupanvirus deep ocean*, and *Moumouvirus*.

### 3.4 Microbial interaction network analysis

**Figure 4:**
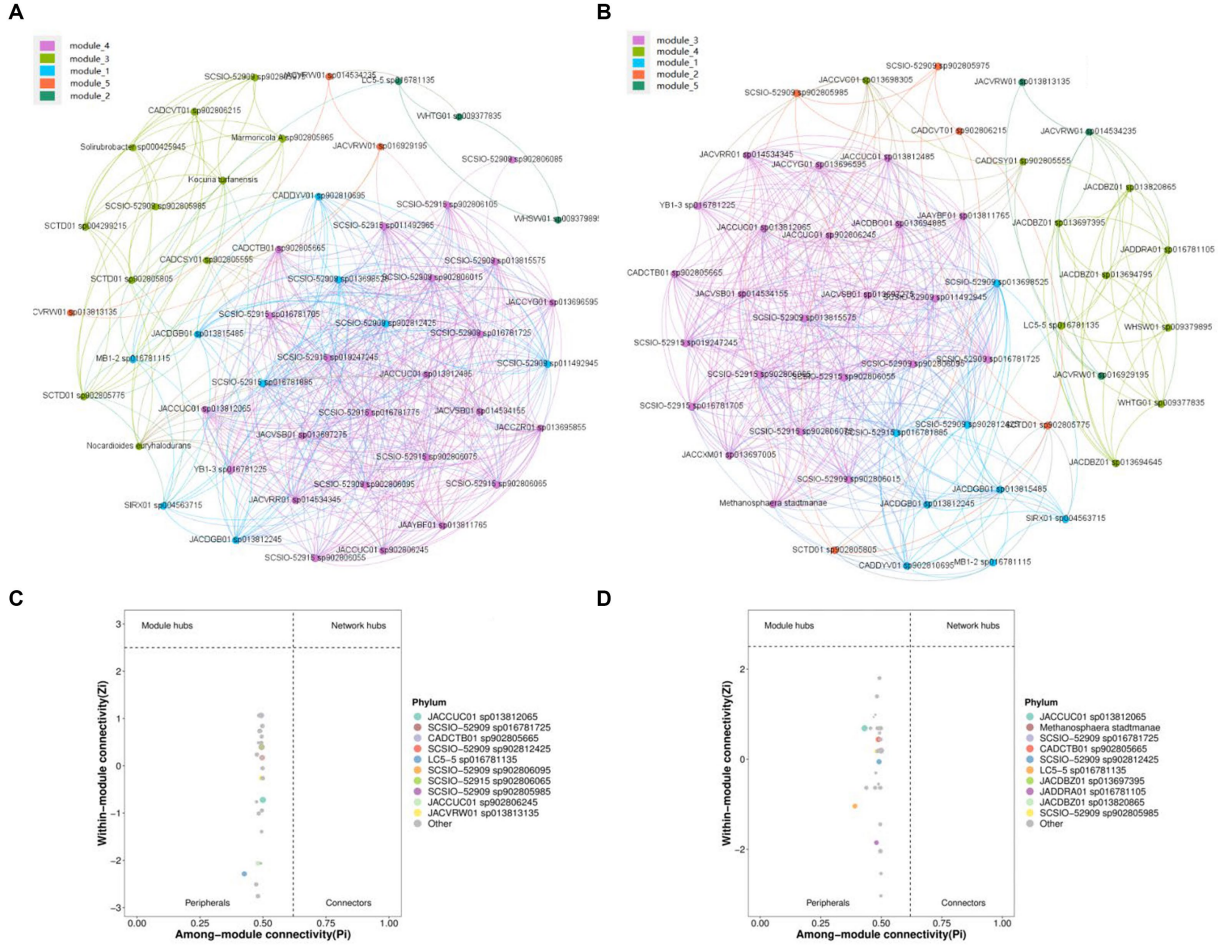
Visualization of the microbial interaction network. **(A)** Co-occurrence patterns of top 50 actinomycetes species in high relative abundance. **(B)** Zipi analysis based on **(A)** network module. **(C)** Co-occurrence patterns of the top 50 microbial species in high relative abundance. **(D)** Zipi analysis based on (c) network module.

The networking between microbes was conducted to investigate the interaction pattern of microbial communities (Faust and Raes, 2012). Spearman correlation was utilized to calculate correlation values among members of the microbial community, resulting in the creation of a correlation matrix (|R| > 0.6, *p* < 0.05). The dominant species seed network was then constructed by extracting the top 100 nodes with average abundance using the igraph package in R. Additionally, the generated gML file could be imported into gephi software for visual representation (Bastian et al., 2009). Two network diagrams were constructed for the top 50 actinomycetes species and the top 50 microbial species with high relative abundance (Figures 4A, C). Each of these diagrams consisted of five modules, with module 1 in Figure 4A and module 5 in Figure 4C containing similar species. The largest modules were module 2 in Figure 4A and module 3 in Figure 4C, which contained 24 and 23 nodes, respectively. However, these modules differed in terms of species composition, with 19 species being common to both and 9 species being unique. Therefore, actinomycete species account for a high proportion of the entire microbial community.

LC5-5 sp016781135, WHSW01 sp009379895, and WHTG01 sp009377835 are the only modules contained in module 2 of Figure 4A. LC5-5 sp016781135, JADDRA01 sp016781105, JACDBZ01 sp013697395, JACDBZ01 sp013820865, WHSW01 sp009379895, WHTG01 sp009377835, JACDBZ01 sp013694795, JACDBZ01 sp013694645, JACCVC01 sp013698305, and CADCSY01 sp902805555 are the only modules contained in module 4 of Figure 4C added into the network.

Nodes in a network can be divided into four parts using Zi and Pi values, namely peripherals, connectors, module hubs, and network hubs (Karimi et al., 2020). Peripherals represent some of the specialists in the microbial network (Deng et al., 2012). Among them, five species of actinomycetes (MB1-2 sp016781115, JACVRW01 sp014534235, YB1-3 sp016781225, JACVRR01 sp014534345, and JACVRW01 sp016929195) interact with species contained in module 4. It is apparent from Figures 4C,D that all the nodes were identified as peripherals (Zi < 2.5 and Pi <0.62).

### 3.5 Analysis of the functional contribution of microbial species

**Figure 5:**
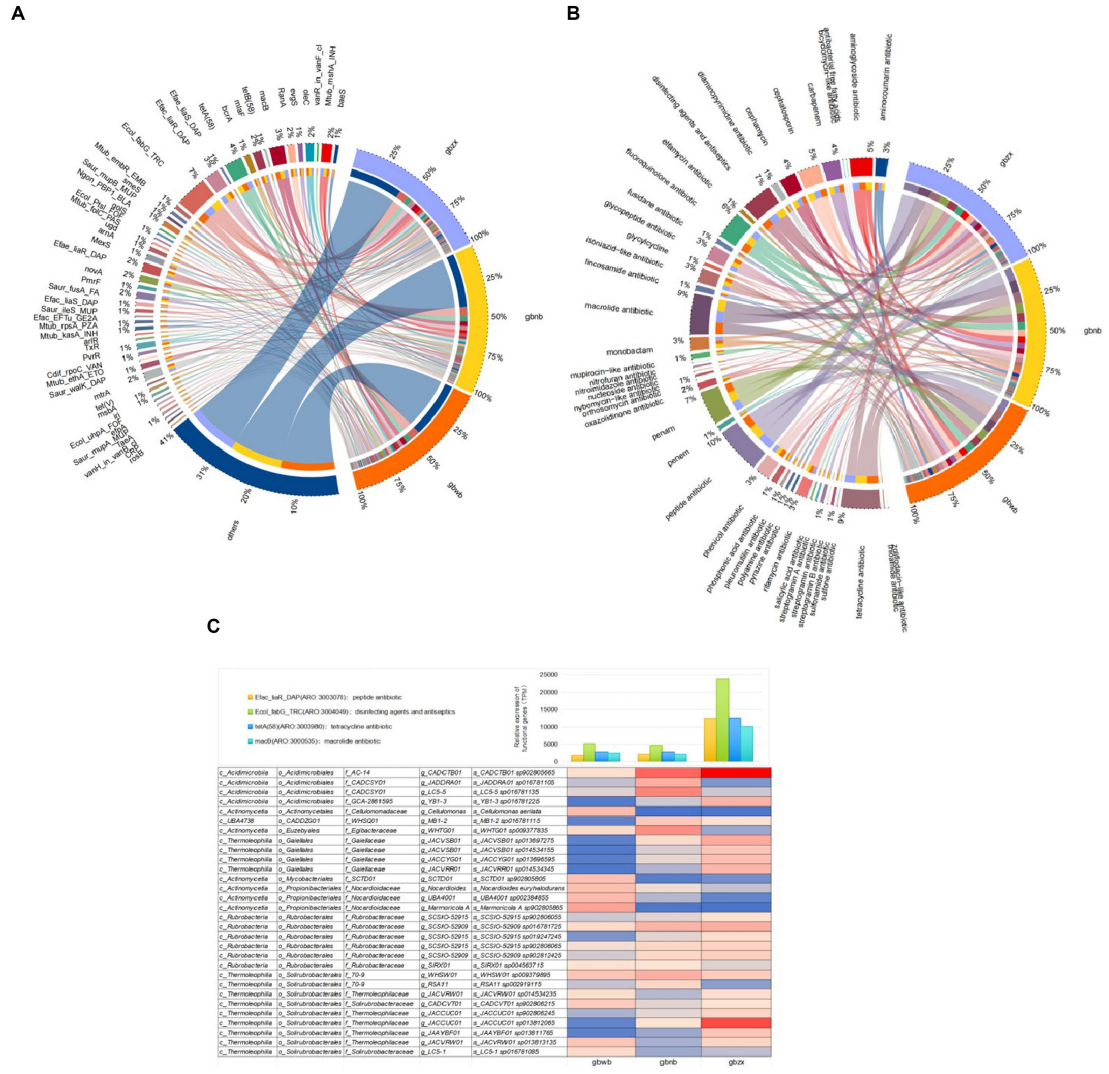
**(A)** Circos diagram of functional gene terms at the level of Drug_Class. **(B)** Circos diagram of functional gene terms at the level of ARO_Name. **(C)** Functional contribution of top 30 microbial species.

The core of the comprehensive antibiotic resistance database (CARD) is the Antibiotic Resistance Ontology (ARO), which is used to associate antibiotic modules with their targets, resistance mechanisms, gene variants, and other information. The gene functions in all samples were annotated and analyzed in the CARD database, resulting in the functional gene abundance table. The relationship between samples and functional genes is illustrated using the Circos diagram (Figures 5A,B). In summary, we found that the functional genes were divided into a total of 44 terms based on the functional level of Drug_Class (Supplementary Table S4). The top 10 terms included peptide antibiotic, macrolide antibiotic, tetracycline antibiotic, penam, disinfecting agents and antiseptics, fluoroquinolone antibiotic, cephalosporin, aminoglycoside antibiotic, carbapenem, and cephamycin (Figure 5A). We found that *Ecol_fabG_TRC*, *Efac_liaR_DAP*, *tet*A (58), *mac*B, *Mtub_mshA_INH*, *ole*C, *Ran*A, *mla*F, *Efae_liaR*_DAP, *nov*A, *Saur_fusA*_FA, and *mtr*A are the top 12 terms according to the functional level of ARO_Name (Figure 5B).

The actinomycetes species corresponding to the top four antibiotic-related genes in relative abundance are displayed in Figure 5C. It can be observed that the top 30 species in relative abundance are all associated with *Ecol_fabG_TRC*, *Efac_liaR_DAP*, *tet*A (58), and *mac*B. These species belong to various taxonomic groups, including five families of the order *Acidimicrobiales* in the class *Acidimicrobiia*, one genus of the family *Egibacteraceae* in the order *Euzebyales* of the class *Acidimicrobiia*, three genera of the *Nocardioidaceae* family in the order *Propionibacteriales* of the class *Actinomycetia*, one family of the order *Mycobacteriales* in the class *Actinomycetia*, four genera of the *Gaiellaceae* family in the order *Gaiellales* of the class *Thermoleophilia*, five genera of the *Thermoleophilaceae* family in the order *Solirubrobacterales* of the class *Thermoleophilia*, two genera of the f_70–9 family in the order *Solirubrobacterales* of the class *Thermoleophilia*, and one genus of the *Solirubrobacterales* family in the order *Solirubrobacterales* of the class *Thermoleophilia*. Additionally, it includes six genera of the *Rubrobacteraceae* family in the order *Rubrobacterales* of the class *Rubrobacteria*.

## 4 Discussion

Alpha and beta diversity analyses were performed to evaluate the variety and abundance of actinomycetes across the three samples. The findings revealed significant variations in the number and diversity of actinomycetes. The exterior of the Gobi exhibited the highest number and diversity, followed by the interior and finally the center (Figure 2A, Supplementary Table S1). Previous literature suggests that actinomycetes primarily exist in soil as spores, requiring nutrients, trace salts, and a neutral pH environment for survival. They can be transported by rainfall, wind, sand, and arthropods. Most actinomycetes are strictly aerobic bacteria (Goodfellow and Williams, 1983). Considering the analysis of soil properties, it is evident that the external soil environment is more conducive to the growth of actinomycetes. Therefore, in studying the diversity of actinomycetes in Gobi soils, it is clear that peripheral soils are more suitable than internal and central soils. In our analysis of CARD data, we observed that the harsh environment in the center of the Gobi desert led to a greater expression of antibiotic genes compared to the periphery and interior regions of the Gobi. Therefore, for the purpose of identifying valuable compounds, we suggest conducting further research on the soil in the central area of the Gobi desert.

Metagenomic studies consistently suggest that there are still many microbial species that need to be thoroughly studied. Out of the top 30 bacteria in terms of relative abundance, 28 belong to the phylum Actinobacteriota (Supplementary Tables S2, S3). This is in good agreement with the previous studies on desert samples (Liu et al., 2018; Leung et al., 2020). Among them, 16 bacteria are from *Rubrobacteraceae*, *Thermoleophilaceae*, *Gaiellaceae*, *Egibacteraceae*, and *Acidimicrobiales*. Previous research revealed that members of the two orders *Gaiellales* and *Rubrobacterales* were so difficult to culture that it severely restricted their function study (Chen et al., 2018, 2021). *Rubrobacter* species are a potential source of bioactive compounds with applications such as radiation-resistant, desiccant-resistant, and enzymatic radical scavengers (Albuquerque et al., 2014). The *Thermoleophilaceae* bacteria can degrade lignin and LDACs (Levy-Booth et al., 2022). Since the first isolation of *Egibaceraceae fam* nov., there have been no studies describing its function, and it has been identified as a halophilic basophilic bacterium from its natural saline-alkaline habitat (Zhang et al., 2016). The causative products of *I. coccineus* YM16-304 can be used in pharmaceutical and chemical synthesis fields (Kim, 2019; Koopmeiners et al., 2017). Additionally, numerous functional studies have been conducted on ferrous oxidation and iron reduction (Melany et al., 2022). The high relative abundance of Halobacteriota archaea suggests their adaptability to extreme environments. Among the species with a high relative abundance of archaea, three phyla (*Methanobacteriota*, *Thermoplasmatota*, and *Thermoproteota*) are related to methanogenesis (Supplementary Table S2). Three types of *Aspergillus* with high abundance were identified in our samples (Supplementary Table S2). Among them, *Aspergillus fumigatus* is a significant pathogen that causes aspergillosis in humans and animals. *Aspergillus terreus*, on the other hand, is known for its ability to produce mevinolin and is currently being commercially produced (Alberts et al., 1980). Although *Aspergillus fumigatus* is closely related to *Aspergillus fumigatus* (Hong et al., 2005), it exhibits different susceptibility to antifungal drugs such as amphotericin B and azoles when compared to *Aspergillus fumigatus* (Alastruey-Izquierdo et al., 2014). Algae crust, primarily composed of desert algae, is commonly found in desert regions.

The current study identified several algae species with relatively high abundance, including Chlorella desiccata, Chloropicon primus, Cladocopium goreaui, Lingulodinium polyedra, *Ostreobium quekettii*, Symbiodinium necroappetens, *Symbiodinium microadriaticum*, Symbiodinium sp. KB8, Trebouxia sp. A1-2, and Pedinophyceae sp. YPF-701. Penicillium nalgiovense and Rhizopus arrhizus are two kinds of food fermentation fungi with relatively high abundance. The presence of plant pathogenic fungi (Pyrenophora tritici-repentis and Rhizoctonia solani) and human pathogenic protozoa (Plasmodium ovale and *Acanthamoeba castellanii*) is abundant. Including mimiviruses, giant viruses, marseilleviruses, pandoraviruses, pithoviruses, faustoviruses, and molliviruses have been increasingly detected in humans and amoebae. This challenges the definition and classification of viruses (Colson et al., 2017). In this study, pandoraviruses and *Acanthamoeba polyphaga* mimivirus were found to be among the top 30 most abundant viruses (Supplementary Table S3). Hepatitis E virus, known as Paslahepevirus balayani, is the main cause of acute viral hepatitis in humans worldwide (Caballero-Gómez et al., 2023). Mammalian orthorubulavirus 5 was detected in healthy civets (He et al., 2022). They may be potential sources of zoonotic pathogens. The results of this study revealed that a high relative abundance of Paslahepevirus balayani and Mammalian orthorubulavirus 5 had a high relative abundance (Supplementary Table S3). Molluscum contagiosum virus is an important human skin pathogen known to cause disfigurement and suffering in children (Chen et al., 2013), and Rotavirus is one of the main pathogens causing infant diarrhea (Gómez-Rial et al., 2020). Molluscum contagiosum virus and Rotavirus were both in high relative abundance in our soil samples (Supplementary Table S2). Additionally, Retroviruses offer promising prospects for gene therapy of human molecular diseases (Twardzik et al., 1982; Frederic Mushinski et al., 1983). Abelson murine leukemia virus was also observed to have a high relative abundance in our samples (Supplementary Table S3).

Our study utilized a metagenomics approach in combination with the CARD database to identify microbial species associated with antibiotics. The results revealed that the top 30 actinomycetes, in terms of relative abundance, also exhibited the highest relative abundance of antibiotic-related genes (refer to Supplementary Tables S3, S4). Peptide antibiotics are active peptides that are widely present in various organisms in nature. They possess diverse activities such as antibacterial, antifungal, antiviral, antiprotozoal, anticancer, and immunomodulatory effects. As a result, they hold potential as a new class of therapeutic drugs alongside traditional antibiotics. Several antimicrobial peptides have already entered the drug development program and have shown promising clinical efficacy with minimal side effects (Lewis, 2020; Upert et al., 2021). Researchers have employed an algorithmic strategy (Torres et al., 2022) to efficiently mine proteomic data, which has opened up new avenues for the discovery of candidate peptide antibiotics. Tetracycline antibiotics, widely used in aquaculture and agriculture, contribute to the development and spread of resistance to such antibiotics (Fang et al., 2020). By modifying the side chain of the linear fusion four-ring scaffold, the second generation of tetracycline antibiotics, represented by doxycycline and minocycline, as well as the third generation, represented by tigecycline, were designed and synthesized (Cui et al., 2022). Apart from their antibacterial effects, tetracyclines have also been explored as cancer therapeutics (Li et al., 2023). Macrolide antibiotics have demonstrated high efficacy in treating respiratory infections. However, they face the challenge of drug resistance. To overcome this, researchers have designed new antibiotics based on the structure of macrolides and their interactions with macromolecular targets (Glanzer et al., 2015; Pichkur et al., 2020). Additionally, researchers have conducted a population-based retrospective cohort study to examine the risk associated with macrolides (Trac et al., 2016). In summary, microbial NPs remain the primary sources for new antibiotic discoveries. More in-depth studies are needed to isolate and culture microbial species and identify antibiotic actives.

## 5 Conclusion

This study aimed to investigate the diversity of actinomycetes in extreme environments. Our findings indicate a higher prevalence of actinomycetes in the soils of the Gobi region. By analyzing the existing literature and genebanks, we made predictions about the metabolites produced by 30 highly abundant actinomycetes. However, further research is needed on those actinomycetes that are challenging to culture. Despite the difficulties, we are confident that we can identify similar species based on the physiological characteristics of known actinomycetes using high-throughput sequencing technology and bioinformatics. This study has enhanced our understanding of microbial diversity in the Gobi region and has provided new methods and theoretical support for future exploration of actinomycetes discovery and NP isolation.

## Data availability statement

The datasets presented in this study can be found in online repositories. The names of the repository/repositories and accession number(s) can be found below: NCBI–SRP467880, BioProject: PRJNA1031080. The data is also available at this link: https://www.ncbi.nlm.nih.gov/sra/PRJNA1031080.

## Author contributions

SY: Writing – original draft, Writing – review & editing. WZ: Writing – original draft. BY: Writing – original draft. XF: Writing – original draft. YL: Writing – original draft. XL: Writing – original draft. QL: Writing – original draft, Writing – review & editing.

## Footnotes

^1^http://kaiju.binf.ku.dk/

^2^https://card.mcmaster.ca/
